# The Impact of Effective Microorganisms (EM) on Egg Quality and Laying Performance of Chickens

**DOI:** 10.1155/2021/8895717

**Published:** 2021-07-07

**Authors:** Alem Tadesse Atsbeha, Teweldemedhn Gebretinsae Hailu

**Affiliations:** Aksum University, College of Agriculture, Department of Animal Sciences, Shire, Ethiopia

## Abstract

Chickens kept under modern production system are very small and contribute less than 2% of eggs and meat production in Ethiopia. In some parts of the country, effective microorganism (EM) has been used as a means of improving egg and meat production. However, there is information gap on the use and effect of EM on egg quality and laying performance of chickens in the local context. This study was conducted in Aksum University's poultry farm located at the main campus in Axum, to evaluate egg laying performance and quality of eggs in layer chickens treated with effective microorganisms in feed and water. In this experiment, 180 pullets of ISA Brown chickens with uniform age and weight were used and managed in a cage system. Chickens were subjected to 4 treatments with 3 replications, and each replication consisted of 15 chickens. Data collection was started at the first egg lay. Data including feed intake, conversion ratio, and age at first laying, laying percentage, and egg quality parameters were collected. Statistical analysis was carried out using JMP. Chickens fed with EM in feed and drinking water had higher egg production percentage. There was a significant difference in egg laying percentage between the treated and control groups (*P* < 0.001). Eggs from chickens given EM in feed and water were 6% heavier in weight than those from control birds. Lower feed daily intake (115.5 gram) and feed conversion rate (2.05) were achieved in chickens treated with EM in feed and water. Significant improvement on egg quality was revealed in chickens that received EM in feed and water. From this experiment, it can be concluded that the use of EM in feed and water improves egg production in layer chickens and therefore recommended for medium-scale poultry farms in Northern Ethiopia.

## 1. Introduction

Poultry rearing is known to create employment, improve human nutrition, generate family income, and plays roles in the social, cultural, and religious lives of societies in developing countries [1, 2]. Ethiopian chickenpopulationis estimated to be 56.07 million, with exotic breed chickens representing 6.45% [3]. Backyard poultry production with indigenous breeds accounts for 98.5% of the national egg production in Ethiopia [4] and contributes significantly to the national economy in general and the rural economy in particular. Tigray region is one of the ajor production areas where backyard poultry production is practiced with a total population of 6.19 million chickens [3]. Almost every family in the rural areas of the country practices traditional chicken production [5]. Despite the large population, per capita egg and chicken meat availability in Ethiopia are very low, estimated to be 0.2 kg and 0.7 kg per person per year, respectively [6]. The traditional system is one of the limiting factors in poultry production. Chickens kept under modern production system are very small and contribute less than 2% of eggs and meat production in the country [7]. However, in the past few years, the percentage of exotic breeds in Ethiopia is increasing from year to year. It was 2.56% in 2013 and became 6.45% in 2018 [3, 8]. Even though the introduction of exotic breeds was carried out as a strategy to improve egg production, poor feed and feeding management are continuing as hindering factors. Different feed additives and supplementations are commonly used to improve the productivity of the sector world-wide. One of the supplementations is the provision of effective microorganism (EM) in feed and/or water, and some farmers in countries like Japan are using them in preference to antibiotics [9]. EM composes of different microbes including photosynthetic bacteria, actinomycetes, yeast, lactobacillus, and fungi [10]. EM was used as a means of improving egg and meat production in chicken farming. According to Xiang et al. (2019) [11], dietary supplementation with probiotics increases egg production and improves feed conversion efficiency. In South Africa, it was used to increase productivity in integrated animal units and poultry farms [12]. When EM is used in rearing sheds, it helps in suppressing diseases and eliminating or controlling ammonia produced by chickens' droppings [13]. As a result, it improves air quality in a poultry house. Using effective microorganisms, the existing low productivity of chickens in Ethiopia can be improved. When a bird has EM in its diet, its immune system [14] and productivity will be boosted [15]. EM can be added to the feed and water so that the beneficial microorganisms will improve the gut flora of the birds, making digestion more efficient and helping to reduce feed costs [16]; hence, profitability could be increased. However, EM technology is new for Tigray and therefore, there was information gap on its application and know how to improve the productivity of chickens through the application of EM in Northern Ethiopia, particularly in Tigray. Therefore, introducing EM technology with different methods of application and evaluating its effect on egg quality and laying performance of Brown *ESA* chickens were the purpose of this study.

## 2. Methodology

### 2.1. Description of the Study Area

This study was conducted at Aksum University's poultry farm, Aksum. The farm has a capacity of 3000 layers in a cage system of poultry production in one room. It is built with a concrete floor, corrugated iron sheet roofing and a wall of concrete block and mesh wire. Feeding and egg collection are carried out manually while watering is an automated nipple system. Aksum is a city located in Central Zone of Tigray about 963 km away to the North from Addis Ababa, Ethiopia, and 202 km from Mekelle, capital city of Tigray regional state, at an average elevation of 2,130 meters above sea level with a geographical coordinates of 14° 7′ 8^″^ N, 38° 43′ 46^″^ E. The average daily temperature of the city is 18.3°C and its average annual rainfall is 652 mm.

### 2.2. Experimental Animals

A total number of 180 pullet chickens of *Brown ISA* with uniform age and weight were bought from Alema Poultry farm in Debrezeyt at the age of 12 weeks. These chickens were divided into four experimental groups, with three replications, and each group contained 15 pullets. All groups were randomly assigned to one of the treatments using a completely randomized design (CRD). Pullets were subjected to the treatments after 2 weeks of their arrival. The experimental room was separated into twelve separate cages (Table 1).

For better biosecurity, the whole house and each pen were cleaned and disinfected with 37% formalin two weeks before the introduction of the pullets.

#### 2.2.1. Treatments

Four treatments were prepared using EM in feed and activated EM solutions as follows:

- CTL: control/without EM
- EM-F: EM in feed
- EM-W: EM in drinking water
- EM-FW: EM in feed and drinking water

EM in feed (solid form) and EM in water (solution form) were prepared following the procedures of the Asia Pacific natural agriculture Network [17]. Daily feed rations were always prepared by adding 2% EM in a solid form before the start of egg laying and 1% after the start. Similarly, 2 ml of activated EM solution was added in a liter of drinking water. Formulated poultry feed was purchased from Ethio-chicken according to their age and production phase. The feed ingredients were analyzed by the feed quality and control groups of Ethio-chicken. All chickens were fed with the formulated diet (Table 2) and provided water *ad libitum* throughout the entire study.

#### 2.2.2. EM Elements

EM is a mixed-cell culture which composes of photosynthetic bacteria, actinomycetes, yeast, lactic acid producing bacteria (lactobacillus), and fermenting fungi produced under the supervision of EMRO Japanese Institute, Okinawa, Japan, as described by Woljeejii Agricultural Industry PLC (WAI), Debre Zeit, Ethiopia.

#### 2.2.3. Feed Ingredients

The composition and ratio of the elements used for the preparation of EM in feed were as EM solution (100 ml), molasses (100 ml), and 10 liter water in 100 kg feed (mesh feed).

The steps applied in the preparation of EM in solid form were dissolving molasses in water to make molasses solution, adding EM in the molasses solution, and spraying the mixture of EM and molasses solution on the feed, and mixing it well. Finally, the mixed feed was packed in air-tight black polyethylene bag for 8 days to be fermented and used.

### 2.3. Management of Experimental Animals

The experimental chickens were managed under a cage system, in the poultry farm of Aksum University. Twelve pens were prepared, and chickens were randomly assigned to be confined within the pen. Chickens were managed based on the breeders' management guide. For all treatment groups, the feed was given in the early morning and late afternoon hours. Since the house was open-sided house, electric florescent light was provided to layers for four hours every evening to fulfill the maximum photoperiod requirement of the layers. Layers were kept until they reach a declining phase of egg laying.

### 2.4. Data Collection

The pullets were subjected to treatment after 2 weeks of their arrival. To estimate the feed intake for each replication and treatment, feed accessible and refusal were weighed and recorded every day throughout the experimental period. Data collection on egg production was started at the first egg laying period. Parameters such as age at first egg laying, number of eggs laid per day, egg laying percentage, egg weight, and mortality of layers were gathered and analyzed according to a data collection schedule set in this research. The eggs were daily collected, weighed with 0.01 g precision, and daily average egg weight recorded. Ten eggs were randomly selected and cracked/broken for internal and external egg quality evaluation from each treatment at the end of peak egg lay time (end of the trial). Yolk and albumen height was measured using a tripod micrometer with a precision of 0.01 mm. Eggshell thickness with shell membrane was measured using digital caliper at the sharp, middle, and blunt parts of the shell, and mean was taken as eggshell thickness. Yolk weight and weight of eggshell with membrane were measured using an electronic balance with a precision of 0.001 g. Albumen weight was determined by subtracting the weight of the yolk and the shell from the weight of the whole egg. Yolk color was measured using a roach color fan, ranged from 1 to 15. Haugh unit was calculated using the formula HU = 100∗log [*h* + 7.57 − 1.7*w*^0.37^] employed by Eisen et al. (1962) [18], where “HU” is the Haugh unit, “*h*” was the height of albumen, and “*w*” was the egg weight.

### 2.5. Data Analysis

Statistical analysis of the primary data was made using JMP. One-way ANOVA was used for continuous data type and was compared by least significance difference. The significance of the number of eggs laid was tested using chi-square statistics. Means were considered statistically different at *P* < 0.05 level of significance.

The mortality rate of the layers was calculated as:

(1) $$\begin{array}{c} \text{Mortality}\%=\frac{\text{Number of birds died}}{\text{Total number of birds}}*100. \end{array}$$

Similarly, the egg laying percentage was calculated as:

(2) $$\begin{array}{c} \text{Egg Production in}\%=\frac{\text{Number of eggs }\left(\text{in numbers}\right)}{\text{Total number of birds}}*100. \end{array}$$

The average daily feed intake was calculated by subtracting the feed left in the feed trough from what was given the previous day and then dividing it by the number of pullets in the replicate. The amount of feed consumed per unit of egg weight was calculated to determine the feed conversion ratio. Feed conversion ratio was calculated by dividing the feed consumed by the unit of egg weight.

## 3. Result and Discussion

### 3.1. Feed Intake and Feed Conversion Rate of Layer Chickens

The average feed consumption of layers in the 21-28 weeks of egg production and peak time (29-39 weeks) is depicted in Table 3. From the beginning of egg laying to the end of the 28^th^ week, daily average feed intake was 115.5 grams for chickens fed EM in feed and water, 115.6 grams for chickens treated with EM in water, 115.9 grams for chickens provided EM in feed, and 116.2 grams for chickens in the control group. The feed intake of chickens fed EM in feed and water was lower than the rest treatments (*P* ≤ 0.0001). Chickens provided EM in water had also lower feed intake (*P* < 0.05) than the chickens provided EM in feed and control groups. In line with this result, previous findings [14, 19] showed a significant difference in feed consumption between the EM-treated and control groups of chickens. Other findings [20] also revealed that dietary supplementation with probiotics not only reduced feed intake but also significantly increased feed conversion. On the contrary, feed intake was higher for broilers fed EM in feed and water during the starter phase [21]. Such difference might be due to the difference in breed and production type of the chickens. In the peak time of egg lay, there was also a significant difference among the treatments in feed intake. Feed to egg conversion ratio of the layers was 2.44 in chickens fed EM in feed, 2.28 in chickens treated with EM in water, 2.05 in chickens provided EM in water and feed, and 2.69 in the control group. The group of chickens treated with EM in feed and water has produced a higher number of eggs with smaller feed consumption. This indicates that chickens under this treatment converted feed to egg more efficiently and produced at a cheaper rate than the control groups. Similarly, Simeamelak et al. (2013) [15] reported that the amount of feed consumed per kg of eggs produced was lowest for groups assigned to treatment containing 4 ml of EM/liter of drinking water. Another report also revealed that birds fed with EM in feed and water required lower feed for a unit increase in weight [22]. With a similar approach, Dahal (2012) [23] found the ratio of feed to the net weight gain of broilers provided EM in water and feed to be higher compared to a control group. This indicated that chickens provided EM in water and/or feed have more efficient utilization of feed than chickens in control. In opposition to the present findings, Wondmeneh et al. (2011) ( [21] suggested that EM has little effect on feed conversion ratio of broilers (FCR).

### 3.2. Age at First Egg Lay

The first egg lay was recorded in chickens provided EM in water and feed at 161 days of age followed by chickens given EM in water at 166 days of age (Table 3). The control chickens started laying at 168 days of age and chickens given EM in feed started at 175 days. The age at first egg lay of this result was faster than the reported ranged between 179 and 186 days [15]. Although the age at first egg lay of the chickens provided EM in feed was observed to be delayed, the results in the remaining other treatments showed that effective microorganisms enhanced early maturity, thereby improving age at first lay. This might be related to the fast growth of the chickens which could be due to the presence of the beneficial microbes in the gut which helps fast utilization of essential nutrients [16]. Dietary probiotic supplementation improved the health and microscopic structure of the ileum and cecum [20].

### 3.3. Mortality Rate

As displayed in Table 3, higher mortality of chickens was observed in chickens provided EM in feed (17.8%) followed by the control group (15.6%). Similarly, Chantsavang and Watcharangkul (1998) [19] reported higher mortality (5.55) in chickens received EM in feed. The remaining two treatment groups of this study (EM treated in water and EM in feed and water) had a relatively lower mortality rate (13.3% each). This result agreed with Gnanadesigan et al. (2014) [16] who reported that mortality ratio decreased in hens fed with EM-treated commercial feed and water. About 66% and 22% of the total mortality was within the first and second months of the commencement of the experiment, respectively. This might be due to the incidence of unidentified disease in the farm in the first month of the chickens' arrival.

### 3.4. Egg Laying Percentage

**Figure 1:**
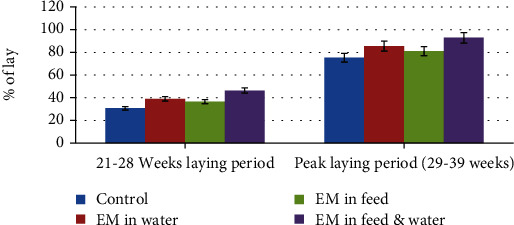
Average daily egg production of chickens in percent treated with EM in feed and water.

Daily average egg production in the 21-28 weeks of laying period and peak egg lay time (29-39 weeks) is illustrated in Figure 1. Daily egg production of the chickens varied with the treatment.

Chickens received EM in feed and EM in drinking water have higher egg production percentage both in the first 21-28 weeks (46.4%) and at peak egg laying period (92.8%) followed by chickens provided EM in water. In line with this result, a higher egg production percentage (83.2%) was reported by [16] in chickens treated with EM in feed and water. In addition, a previous finding [24] reported that hens given EM-treated feeds have a higher difference in egg production. A marked difference (*P* < 0.01) in egg production of Rhode Island Red layers was also reported [15] in a group that received 4 ml of EM/liter of drinking water. This might be due to the beneficial effect of the effective microorganisms in the gut of the chickens by suppressing pathogenic bacteria and facilitating digestion and absorption of nutrients. Moreover, others [25] also confirmed that the use of EM at 1% to have the best effect in reducing the growth of pathogenic bacteria. In contrast to the current result, Fathiet al. (2018) [26] reported no significant difference in egg production performance among the different dietary treatments.

As shown in Table 4, the average numbers of eggs per day produced in the first 21-28 weeks and at the peak laying period were 18.4 and 36.2, respectively, in chickens received EM in water and feed. These results were significantly higher than the eggs produced by the control group (*P* < 0.05) both at the first 21-28 weeks and peak egg laying time. This might be due to increasedflora of the digestive tract, which facilitates feed digestion, absorption, and conversion of feed to egg. The current result is in agreement with the finding of Naqvi et al. (2000) [24] who reported that egg number was significantly greater in hens given feed containing 1 and 2% EM than the control hens. Similarly, addition of EM with different levels improved egg number during the laying period [27].

### 3.5. Internal and External Egg Quality

The average egg weight in the first 21-28 weeks of the egg laying period was 59.1 grams in chickens received EM in water, 58.3 grams in chickens received EM in feed, 60.9 grams in chickens received EM in water and feed, and 57.2 grams in chickens received control feed (Table 5). Egg weight varied with the treatments in the peak egg laying period (29-39 weeks). There was a significant difference (*P* < 0.0001) in egg weight between chickens provided EM in feed and water and the control group both in the first 21-28 weeks and in the peak egg lay time. In line with this, Gnanadesigan et al. (2014) [16] reported a maximum egg weight (61.6 ± 5.83 g) in a group of EM-treated layers in feed and drinking water. Unlike the current result, El-Deep et al. (2011) [27] reported that the average weight of eggs from EM-treated chickens was found similar to the control groups. Other findings [28] showed that EM has no significant effect on egg weight. This variation could be due to differences in breed, feed composition, and environmental factors.

Internal and external egg quality parameters are illustrated in Table 6. The overall mean of yolk height, albumen height, eggshell thickness, eggshell weight, yolk weight, albumen weight, and Haugh unit were 12.86 ± 0.2 mm, 5.54 ± 0.1 mm, 0.368 ± 0.009 mm, 5.73 ± 0.07 g, 15.68 ± 0.18 g, 37.76 ± 0.15 g, and 73.15 ± 0.73, respectively, and average yolk color was 6.53 ± 0.19. The overall mean yolk height in this study was slightly lower than a previous report [15], whereas yolk weight, albumen height, and albumen weight in their report were by far smaller than the current result. Shell weight in this finding was lower than former reports[16, 19] but shell thickness was higher than the reported 1.44 mm in chickens provided EM in feed and water [16]. The application of EM in feed and water has shown significant (*P* < 0.05) effect on yolk height, albumen height, eggshell weight, albumen weight, and Haugh unit of eggs. This might be due to the improvement in the assimilation of nutrients such as calcium, phosphorus, carotenoid, and albumen in the serum of layers, in the digestion system [26, 29]. Moreover, these authors stated that the acidic environment, created due to the presence of probiotic, facilitates the ionization of minerals, which is essential for the absorption of calcium and phosphorus. This result supported the finding of El-Deep et al. [27] which reported that feeding birds on diets supplemented with different levels of EM and zinc bacitracin significantly affected egg quality traits. This also agreed with Gnanadesigan et al. [16] that shell weight, yolk weight, and Haugh units varied between the chemical and EM-treated layers. Likewise, shell thickness and weight of eggshell were significantly improved in laying hens fed a diet containing probiotics [26]. On contrary, no significant effect on most of the egg quality traits was reported by others [20, 28]. A higher numeric value of yolk color was found in chickens supplemented with EM in feed and water than a group that received the control diet although it is not significantly varied (Table 5). Similarly, a research [19] reported that the addition of EM in feed and water improved the yellow color of egg yolk in laying chickens. The average yolk color found in this study was 6.53 ± 0.19, which is lower than the findings of many others [20, 26, 29].

### 3.6. Budget Implication of Using EM

As discussed in previous sections, the addition of EM in drinking water and feed showed a significant increase in egg production both in the number of eggs and egg weight. The age at first egg lay of the chickens treated with EM was also faster than the control groups. These imply that the use of EM in feed and water improves egg production performance in layer chickens. Taking constant all inputs (feed, water, labor, etc…), the only added input cost was for the purchasing and transportation of EM and molasses. The price of EM and molasses including transport was 450 Ethiopian Birr (ETB) per liter whereas the price of an egg was 5.50 ETB (Table 6). One liter of EM is enough to make one tone of EM-treated feed (bokashi), and only 1-2% of their daily ration was sufficient to mix the bokashi with the feed. In addition, 0.25 ml to 0.5 ml of EM is used per one liter of drinking water. Eventually, the addition of EM in feed and water resulted in an increment of 7.6 eggs per day, which brings about 40 ETB per day per treatment group. Considering the cost of EM and the price of an egg, the profitability of adding EM to poultry feed and water is analyzed and found to be profitable. A significant economic implication after the use of 4 ml of EM/liter of drinking water was also found by Simeamelak et al. [15]. Similarly, cost analysis of EM treatment [16] showed a tremendous gain over the use of other chemicals. Inclusion of 5% EM in feed (bokashi) was also found profitable in lambs fed low protein diet [30]. The cost of production versus net income is illustrated in Table 7.

Moreover, the difference in weight of the egg has also great value in market price because the larger the size of the egg, the higher is its market price. Overall, it is possible to get at least 40.30 ETB per day additional net profit from 45 chickens by adding EM in their feed and drinking water. Moreover, according to the result in this study, the inclusion of EM in feed and water enhanced egg quality. As a result, the improved quality can increase the market value and demand for the eggs. Hence, recently considerable attention has been paid to the quality and safety of food throughout the world.

## 4. Conclusion

The use of effective microorganisms in poultry feed and water can improve the performance of layer chickens and increase internal and external egg quality. Therefore, the application of EM in feed and water is found to be profitable and recommended for medium-scale poultry farms in Northern Ethiopia. However, EM in water was more applicable, easy, and effective for small-scale poultry producers.

## Figures and Tables

**Table 1:** Experimental cages and experimental treatments.

Replicate	Control group	Experimental group		
	T1(CTL)	T2 (EM-F)	T3 (EM-W)	T4 (EM-FW)
R1	15	15	15	15
R2	15	15	15	15
R3	15	15	15	15
Total	45	45	45	45

**Table 2:** Ingredients (% air-dry basis) of the feed.

Raw materials	Inclusion rate
Maize^1^	57.4%
Soybean meal^2^	22.5%
Wheat bran^3^	8.8%
Limestone^4^	8.6%
Layer premix^5^	2.5%
Toxin binder	0.2%
Total	100%

^1^8% CP, ^2^47% CP, ^3^16 CP, ^4^35% calcium, ^5^2.5% vitamins and minerals, Source: Ethio-chickens (feed supplier).

**Table 3:** Daily feed intake (gram), feed conversion ratio, and mortality in layers treated with EM.

	CTL (mean ± SE)	EM-F (mean ± SE)	EM-W (mean ± SE)	EM-FW (mean ± SE)	O. mean (mean ± SE)	*P* value
Feed intake						
In 21-28 weeks (g)	116.24 ± 0.04^a^	115.9 ± 0.03^b^	115.58 ± 0.02^c^	115.5 ± 0.02^c^	115.8 ± 0.02	<0.0001
In peak laying (29-39 weeks) (g)	116.42 ± 0.05^a^	116.12 ± 0.06^b^	115.97 ± 0.05^bc^	115.86 ± 0.04^c^	116.09 ± 0.03	<0.0001
Feed conversion ratio at peak lay	2.69	2.44	2.28	2.05		
Age at 1^st^ egg lay (days)	168 ± 0.24	175 ± 0.24	166 ± 0.14	161 ± 0.16	166.18 ± 0.7	<0.0001
Mortality in %	15.6	17.8	13.3	13.3		

Different superscripts across rows are significant at *P* < 0.05. CTL: control; EM-F: EM in feed; EM-W: EM in water; EM-FW: EM in feed and water; SE: standard error mean.

**Table 4:** Daily egg production of chickens treated with EM in feed and water.

	CTL (mean ± SE)	EM-F (mean ± SE)	EM-W (mean ± SE)	EM-FW (mean ± SE)	O. mean (mean ± SE)	*P*
In 21-28 weeks of lay	11.9 ± 1.1^c^	14.1 ± 1.5^bc^	15.5 ± 1.3^ab^	18.4 ± 1.2^a^	14.9 ± 0.6	0.0038
In peak egg lay (29-39 weeks)	28.6 ± 0.7^c^	30.0 ± 0.6^c^	33.3 ± 0.9^b^	36.2 ± 0.4^a^	32 ± 0.5	<0.0001

CTL: control; EM-F: EM in feed; EM-W: EM in water; EM-FW: EM in feed and water; SE: standard error mean.

**Table 5:** Average egg weight of chickens treated with EM (effective microorganisms).

	CTL (mean ± SE)	EM-F (mean ± SE)	EM-W (mean ± SE)	EM-FW (mean ± SE)	O. mean (mean ± SE)	*P*
In 21-28 weeks	57.2 ± 0.4^b^	58.3 ± 0.4^b^	59.1 ± 0.6^ab^	60.9 ± 0.6^a^	58.9 ± 0.3	<0.0001
In peak time	57.5 ± 0.4^c^	58.8 ± 0.6^bc^	59.4 ± 0.2^b^	61.0 ± 0.5^a^	59.1 ± 0.2	<0.0001
Mean	57.4 ± 0.4^c^	58.6 ± 0.6^bc^	59.3 ± 0.5^b^	60.9 ± 0.6^a^	59.2 ± 0.2	<0.0001

Different superscripts across rows are significant (*P* < 0.05). CTL: control; EM-F: EM in feed; EM-W: EM in water; EM-FW: EM in feed and water; SE: standard error.

**Table 6:** The effect of EM supplementation on internal and external egg quality.

Quality parameters	CTL (mean ± SE)	EM-F (mean ± SE)	EM-W (mean ± SE)	EM-FW (mean ± SE)	O. mean (mean ± SE)	*P* value
Yolk height (mm)	11.65 ± 0.25^b^	12.05 ± 0.34^b^	13.57 ± 0.19^a^	14.16 ± 0.09^a^	12.86 ± 0.2	0.0001
Albumen height (mm)	5.01 ± 0.16^c^	5.35 ± 0.13^bc^	5.65 ± 0.15^ab^	6.15 ± 0.14^a^	5.54 ± 0.1	0.0001
Shell thickness (mm)	0.346 ± 0.005	0.35 ± 0.005	0.371 ± 0.005	0.404 ± 0.034	0.368 ± 0.009	0.0948
Shell weight (g)	5.4 ± 0.04^b^	5.57 ± 0.09^b^	5.79 ± 0.12^ab^	6.18 ± 0.15^a^	5.73 ± 0.07	0.0001
Yolk weight (g)	15.13 ± 0.37	15.26 ± 0.33	16.03 ± 0.38	16.29 ± 0.29	15.68 ± 0.18	0.0563
Albumen weight (g)	36.98 ± 0.3^c^	37.97 ± 0.28^ab^	37.58 ± 0.2b^c^	38.53 ± 0.24^a^	37.76 ± 0.15	0.0008
Haugh unit (HU)	69.43 ± 1.4^c^	71.88 ± 1.07^bc^	74.03 ± 1.16^ab^	77.25 ± 1.05^a^	73.15 ± 0.73	0.0004
Yolk color	6.2 ± 0.29	6.6 ± 0.43	6.5 ± 0.43	6.8 ± 0.36	6.53 ± 0.19	0.7315

Different superscripts across rows are significant (*P* < 0.05). CTL: control; EM-F: EM in feed; EM-W: EM in water; EM-FW: EM in feed and water; SE: standard error mean.

**Table 7:** Profit analysis of egg production of chickens treated with EM in feed and water.

Number of birds/treatment	45	
*Costs*		
Cost of EM, molasses, and transport	1.45	ETB for 45 hens/day
Communication	0.05	ETB for 45 hens/day
Total cost	1.50	ETB for 45 hens/day
*Revenues*		
Additional egg produced in compared to the control groups	7.60	
Egg weight difference	3.57	Gram
Price of egg	5.50	ETB/egg
Gross income from additional eggs/day	41.80	ETB
Net income or profit/day	40.30	ETB

NB. 1 USD was exchange with 33 ETB during the research period.

## Data Availability

The datasets used for the conclusion of the current study are included in the article in tables and figures and also available from the corresponding author on reasonable request.

## References

[1] Tadelle D., Ogle B. (2001). Village poultry production system in the central high lands of Ethiopia. *Tropical Animal Health and Production*.

[2] Halima M., Neser F., De-Kock A., Van Marle-Koster E. (2009). Study on the genetic diversity of native chickens in North-West Ethiopia using microsatellite markers. *African Journal of Biotechnology*.

[3] Central Statistical Agency (CSA) (2018). Agricultural sample survey; report on livestock and livestock characteristic. *Statistical Bulletin*.

[4] The T. D. (2003). Role of scavenging poultry in integrated farming systems in Ethiopia. *Livestock Feed Resources within Integrated Farming Systems*.

[5] Solomon D. (2003). *Growth Performance and Survival of Local and White Leghorn Chickens Under Scavenging and Intensive Systems of Management in Ethiopia*.

[6] Tafere K., Worku I. (2012). Consumption patterns of livestock products in Ethiopia: elasticity estimates using HICES (2004/05) data, ESSP II Working Paper. http://www.ifpri.org/sites/default/files/publications/esspwp38.pdf.

[7] Gebre M. (2018). Poultry production, consumption, marketing and associated challenges in Ethiopia. *British Journal of Poultry Sciences*.

[8] Central Statistical Agency (CSA) (2013). Agricultural sample survey; report on livestock and livestock characteristic. *Statistical Bulletin*.

[9] Nava G. M., Bielke L. R., Callaway T. R., Castañeda M. P. (2005). Probiotic alternatives to reduce gastrointestinal infections: the poultry experience. *Animal Health Research Reviews*.

[10] Towett G. (2016). What are effective microorganisms? Permaculture research institute. https://www.permaculturenews.org.

[11] Xiang Q., Wang C., Zhang H., Lai W., Wei H., Peng J. (2019). Effects of different probiotics on laying performance, egg quality, oxidative status, and gut health in laying hens. *Animals*.

[12] Hanekon D., Prinsloo J., Schoonbee H. A comparison of the effect of anolyte and EM on the faecal bacteria loads in the water and on fish produced in pig com fish integrated production units. 2001.

[13] Li W., Ni Y. (2008). Use of effective microorganisms to suppress malodors of poultry manure. *Journal of Crop Production*.

[14] Jwher Dh M., Abd S. K., Mohammad A. G. (2013). The study of using effective microorganisms (EM) on health and performance of broiler chicks. *Iraqi Journal of Veterinary Sciences*.

[15] Simeamelak M., Solomon D., Taye T. (2013). The effect of effective microorganisms on production and quality performance of Rhode Island red layers. *International Journal of Livestock Production*.

[16] Gnanadesigan M., Isabella S., Saritha P., Ramkumar L., Manivannan N., Ravishankar R. (2014). Quality evaluation of egg composition and productivity of layers in EM (effective microorganisms) treatments: a field report. *Egyptian journal of basic and applied sciences*.

[17] Asia-Pacific Natural Agriculture Network-APNAN (1995). *EM application manual*.

[18] Eisen E. J., Bohren B. B., Mckean H. E. (1962). The Haugh unit as a measure of egg albumen quality^1^. *Poultry Science*.

[19] Chantsavang S., Watcharangkul P. Influence of EM on the quality of poultry products.

[20] Xiang Q., Wang C., Zhang H., Lai W., Wei H., Peng J. (2019). Effects of different probiotics on laying performance, egg quality, oxidative status, and gut health in laying hens. *Animals*.

[21] Wondmeneh E., Getachew T., Dessie T. (2011). Effect of effective microorganisms (EM®) on the growth parameters of Fayoumi and Horro chicken. *International Journal of Poultry Science*.

[22] Esatu W., Adey M., Tadelle D. (2011). Effect of effective microorganisms on growth parameters and serum cholesterol levels in broilers. *African Journal of Agricultural Research*.

[23] Dahal B. K. Effective microorganism (EM) for animal production. http://www.infrc.or.jp/knf/6th_Conf_S_3.html.

[24] Naqvi Z. H., Mushtaq-ul-Hassan M., Chaudry Z., Akrem M., Ahmad R. (2000). Effect of effective microorganisms (EM4) on health of layers. *Pakistan Journal of Biological Sciences*.

[25] Hamad M. A., Hussein S. A., Mahmmoud E. N., Al-Aalim A. M. (2020). The inhibitory role of effective microorganisms on the growth of pathogenic bacteria. *Iraqi Journal of Veterinary Sciences*.

[26] Fathi M., Al-Homidan I., Al-Dokhail A., Ebeid T., Abou-Emera O., Alsagan A. (2018). Effects of dietary probiotic (Bacillus subtilis) supplementation on productive performance, immune response and egg quality characteristics in laying hens under high ambient temperature. *Italian Journal of Animal Science*.

[27] El-Deep M. H., Amber K., Sayed M. A. M. The effects of supplementation of effective microorganisms on egg production traits, quality parameters and chemical analysis during the late laying period in hens. 2011. In animal hygiene and sustainable livestock production.

[28] Chotisasitorn S., Chantsavang S., Attamangkune S., Plaiboon A. (1997). Using an effective microorganism supplementation in layers. *Kasetsart Journal (Natural Science)*.

[29] Xu C. L., Ji C., Ma Q., Hao K., Jin Y., Li K. (2006). Effects of a dried Bacillus subtilis culture on egg quality. *Poultry Science*.

[30] Deribe G., Shimelis M., Fitsum T., Melese Y., Shewangizaw W., Tewodros G. (2017). Effect of inclusion rate of effective microbes (Em) on growth rate of lambs fed low protein diet. *Biomedical Journal of Scientific & Technical Research*.

